# Notes and News

**Published:** 1895-11-30

**Authors:** 


### NOTES AND NEWS.

(Collected and Communicated.)

Sir Dyce Duckworth presided at the dinner of past and present students of the National Dental Hospital last week. During the evening prizes were presented to the successful students.

A list of the subscribers in Great Britain to the John S. Billings testimonial has now been circulated. It amounts to about $2,200, and it is remarkable that, with the exception of this journal, no other British publication has shown its sense of indebtedness to the work of Dr. John S. Billings by supporting this movement. Yet there is not a scientific journal of any importance published which does not owe an immense debt of gratitude to Dr. John S. Billings, or could make its columns half as accurate and exhaustive when treating of many subjects as they are at present except by the aid of his index catalogue and other publications. Dr. Billings has now retired, and such absence of recognition on the part of medical and scientific journalists in this country is another proof that gratitude is a lively expectation of favours to come.

In The Hospital for April 20th, 1895, page 49, we called attention to "An Old-Fashioned Election: How not to elect a house-surgeon." We at the same time suggested a new system for electing the medical and other officers to the governors of the Northampton County Infirmary. Our recommendations have been approved by the governors, and the first election under the new rules will take place to-day (Saturday). It may be remembered that our proposal was to select by ballot an Election Committee of one hundred governors from the whole body. With cruel irony the ballot box has placed our name on the Election Committee, and we have perforce to go to Northampton in order to record our vote. This is the latest and not the least amusing instance of an engineer being hoist with his own petard.

Dr. Cartwright Wood has received a grant of £100 from the committee of the conjoint laboratories of the Colleges of Physicians and Surgeons, for his investigations as to rapid methods of obtaining diphtheria anti-toxin serum.

At the dinner in aid of the building fund of the Royal Hospital, Richmond, presided over by His Royal Highness the Duke of Cambridge, the Secretary stated that of the £1,500 required to complete the extensions £1,000 had been subscribed, whereupon Mr. Andrew Pears, J.P., of Isleworth, rose and offered the remaining sum of £500, in addition to £50 donation previously made by him.

The Emperor Francis has issued a decree which settles the status of women at the Hungarian Universities. They are now to be admitted to the medical and philosophic faculties, and thus enabled to become chemists, doctors, and professors. It seems hardly necessary to have added the saving clause which renders it necessary for permission to be first obtained from the Minister of Justice.

It is gratifying to realise how much the special efforts made on behalf of the hospitals this year is appreciated by the authorities of the various institutions. Amongst other expressions of recognition which we have already received of the part taken by The Hospital in the matter, we must especially mention the gracious resolution, containing hearty thanks, passed at a meeting of the House Committee of the London Hospital, conveyed to us through the chairman.

Much indignation has been aroused by the announcement that the Trinity Brethren had ordered the destruction of their almshouses in the Mile End Road, rather than expend money on necessary repairs. It appears that the lacking sum is about £150, and the readers of the *Daily Chronicle* have already subscribed that amount to save the interesting building, and the amount has been conveyed to the Brethren. The almshouses were erected in the time of James II., after the designs of Sir Christopher Wren. The structure is quaint and beautiful, and one of the few now remaining of its kind in London.

The Royal National Hospital for Consumption on the separate principle at Ventnor has received a significant and powerful accession of strength by the acceptance of the office of "vice-president" by the following noblemen and gentlemen, well known for their benevolent interest in poor consumptives, namely: The Duke of Newcastle, the Duke of Westminster, Earl Fitzwilliam, Viscount Powerscourt, the Right Rev. the Lord Bishop of Winchester, the Right Hon. Sir John Mowbray, Bart., M.P., Mr. Albert Brassey, Mr. Frederick Colman, J.P., Mr. N. R. Humphrys, Mr. F. D. Mocatta, Mr. Howard Morley, Colonel Charles Seely, Mr. Henry Thompson, Mr. Augustus Thorne, J.P., and Mr. John Watney. Every bed, as usual, is occupied, while there are 143 men and women, approved candidates, waiting for admission as vacancies may occur. Additional funds are much needed in view of liabilities due.

